# Correction for Raines et al., “Nanoparticle delivery of Tat synergizes with classical latency reversal agents to express HIV antigen targets”

**DOI:** 10.1128/aac.00817-25

**Published:** 2025-07-21

**Authors:** Samuel L. M. Raines, Shane D. Falcinelli, Jackson J. Peterson, Ellen Van Gulck, Brigitte Allard, Jennifer Kirchherr, Jerel Vega, Isabel Najera, Daniel Boden, Nancie M. Archin, David M. Margolis

## AUTHOR CORRECTION

Volume 68, no. 7, e00201-24, 2024, https://doi.org/10.1128/aac.00201-24. Page 17, Acknowledgments, paragraph 1: "…(CARE)] to D.M.M." should read "…(CARE)] and NIH UM1AI169633 to D.M.M."

Page 18, Funding, line 1: Grant NIH UM1AI169633 should be added.

